# Overweight/Obesity-related microstructural alterations of the fimbria-fornix in the ABCD study: The role of aerobic physical activity

**DOI:** 10.1371/journal.pone.0287682

**Published:** 2023-07-12

**Authors:** Jiyoung Ma, Erin C. McGlade, Rebekah S. Huber, In Kyoon Lyoo, Perry F. Renshaw, Deborah A. Yurgelun-Todd

**Affiliations:** 1 Department of Psychiatry, University of Utah School of Medicine, Salt Lake City, Utah, United States of America; 2 Diagnostic Neuroimaging Laboratory, Huntsman Mental Health Institute, University of Utah School of Medicine, Salt Lake City, Utah, United States of America; 3 George E. Wahlen Department of Veterans Affairs Medical Center, VA VISN 19 Mental Illness Research, Education and Clinical Center, Salt Lake City, Utah, United States of America; 4 Ewha Brain Institute, Ewha W. University, Seoul, South Korea; Inner Mongolia University of Science and Technology, CHINA

## Abstract

Childhood overweight/obesity has been associated with negative consequences related to brain function and may involve alterations in white matter pathways important for cognitive and emotional processing. Aerobic physical activity is a promising lifestyle factor that could restore white matter alterations. However, little is known about either regional white matter alterations in children with overweight/obesity or the effects of aerobic physical activity targeting the obesity-related brain alterations in children. Using a large-scale cross-sectional population-based dataset of US children aged 9 to 10 years (*n* = 8019), this study explored the associations between overweight/obesity and microstructure of limbic white matter tracts, and examined whether aerobic physical activity may reduce the overweight/obesity-related white matter alterations in children. The primary outcome measure was restriction spectrum imaging (RSI)-derived white matter microstructural integrity measures. The number of days in a week that children engaged in aerobic physical activity for at least 60 minutes per day was assessed. We found that females with overweight/obesity had lower measures of integrity of the fimbria-fornix, a major limbic-hippocampal white matter tract, than their lean peers, while this difference was not significant in males. We also found a positive relationship between the number of days of aerobic physical activity completed in a week and integrity measures of the fimbria-fornix in females with overweight/obesity. Our results provide cross-sectional evidence of sex-specific microstructural alteration in the fimbria-fornix in children with overweight/obesity and suggest that aerobic physical activity may play a role in reducing this alteration. Future work should examine the causal direction of the relationship between childhood overweight/obesity and brain alterations and evaluate potential interventions to validate the effects of aerobic physical activity on this relationship.

## Introduction

Childhood obesity is a major pediatric health concern in the United States, with more than a third of children and adolescents considered overweight or obese [1]. In addition to causing physical health problems, overweight/obesity in children has been associated with a number of negative consequences related to brain function, including low academic performance and poor psychological well-being [2–5]. Without intervention, childhood overweight/obesity may persist into adulthood and increase the risk of developing neuropsychiatric disorders, such as depression and early-onset dementia [5, 6]. There is little information regarding regional brain alterations associated with childhood overweight/obesity, making it difficult to understand the brain mechanisms that may underlie the development of mental health-related outcomes in children with overweight/obesity, thus limiting the ability to establish advanced therapeutic strategies.

Previous studies demonstrated that adiposity negatively affects white matter microstructure through multiple pathways, including the upregulation of neuroinflammation, insulin resistance, and vascular dysfunction [7, 8]. One well-documented finding from animal and adult human studies is reduced plasticity in the hippocampal circuits [8–11]. Recent animal studies have shown that a high-fat diet stimulates neuroinflammation, decreasing brain-derived neurotrophic factor (BDNF) levels, and results in impaired axonal growth in the hippocampal circuits [8]. It is noteworthy that neuroimaging studies in adults have also indicated that the fimbria-fornix, a major limbic-hippocampal white matter tract, is more likely to be vulnerable to obesity-related metabolic complications than other white matter tracts [9–11]. Interestingly, it has also been demonstrated that lesions in the hippocampal circuits influence appetite behavior and subsequent weight gain in animals [12], suggesting that microstructural alterations in this brain region could precede weight gain and may be a target for obesity prevention as well.

As a major projection and commissure tract leading out of the hippocampus to other limbic structures, the fimbria-fornix plays an important role in memory and affective processing [13, 14]. In addition to the known impact of sociocultural and interpersonal factors (e.g., weight stigma, lower self-esteem, and body image concern) in children with overweight/obesity [15], microstructural alterations of the fimbria-fornix in relation to overweight/obesity may also be a possible brain mechanism involved in the development of cognitive and emotional problems in this population.

Efforts to investigate white matter alterations in children with overweight/obesity have increased in recent years. However, while some studies have shown global decreases in white matter volume or integrity in children with obesity [16, 17], findings on regional alterations are few and inconsistent, potentially due to small sample sizes and varying sample characteristics. Although neuroimaging studies in adults have reported evidence supporting the obesity-related regional white matter alterations, detecting such associations may be more challenging in children for two main reasons. First, if we assume the adverse effects of adiposity on the brain, such effects may be less accumulated in children than in adults [7], suggesting that the effect sizes may be relatively small in children. Further, the brain undergoes profound development throughout childhood, making it difficult to isolate the obesity-related alterations in the brain from the age-related variations when the sample includes a broad age range. To precisely characterize the obesity-related regional brain alterations in children, a study design may require both a sufficient sample size to detect the small effects as well as a narrow age span rather than a broader age range to minimize possible age-related changes.

Aerobic physical activity is a safe and easily accessible intervention for improving brain plasticity. Previous studies showed that aerobic physical activity/exercise has a positive effect on neuroinflammation, cerebrovascular function, and neurotrophic factor levels [18, 19]. Based on these previous findings, it is likely that dysfunctions of the hippocampus and adjacent brain pathways may have a high potential for recovery after engaging in aerobic physical activity, possibly due to their high plasticity [19, 20]. However, it remains unclear the extent to which aerobic physical activity would impact white matter integrity in children with overweight/obesity. Examining such relationships may help guide public health intervention strategies.

Restriction spectrum imaging (RSI) is an advanced diffusion modeling technique that enables the characterization of water diffusion undetectable by traditional diffusion tensor imaging (DTI) techniques [21]. Traditional DTI techniques can calculate within-voxel diffusion but are not well equipped to resolve sub-voxel complexities, such as crossing or bending fibers [22]. Unlike traditional DTI, RSI utilizes multi-shell diffusion-weighted imaging data obtained across a broader range of b-values in multiple directions to separate water diffusion within the restricted compartment from the hindered compartment across different tissue geometries. Thus, RSI provides improved quantification of white matter microstructural characteristics, which accounts for within-voxel crossing and bending fibers, and may be less affected by partial volume effects [21]. RSI metrics may be more sensitive in detecting microstructural properties of the developing brain when compared with traditional DTI measures [23] and provide a novel opportunity to study white matter compromise in children with overweight/obesity.

In this study, we aimed to examine (1) the association between childhood overweight/obesity and limbic white matter microstructural characteristics and (2) whether aerobic physical activity reduces the white matter microstructural alterations in children with overweight/obesity. We primarily focused on the fimbria-fornix since this region has been suggested to be susceptible to overweight/obesity [8–11]. We utilized data from the Adolescent Brain Cognitive Development (ABCD) Study^®^, a large-scale population-based cohort study of children aged 9 to 10 years recruited from 21 data collection sites across the United States [24, 25]. This dataset provides several advantages in addressing our research questions, including well-powered analyses of white matter microstructures in relation to overweight/obesity and aerobic physical activity, given the unprecedented sample size with a narrow age range.

## Materials and methods

### Participants

**Fig 1:**
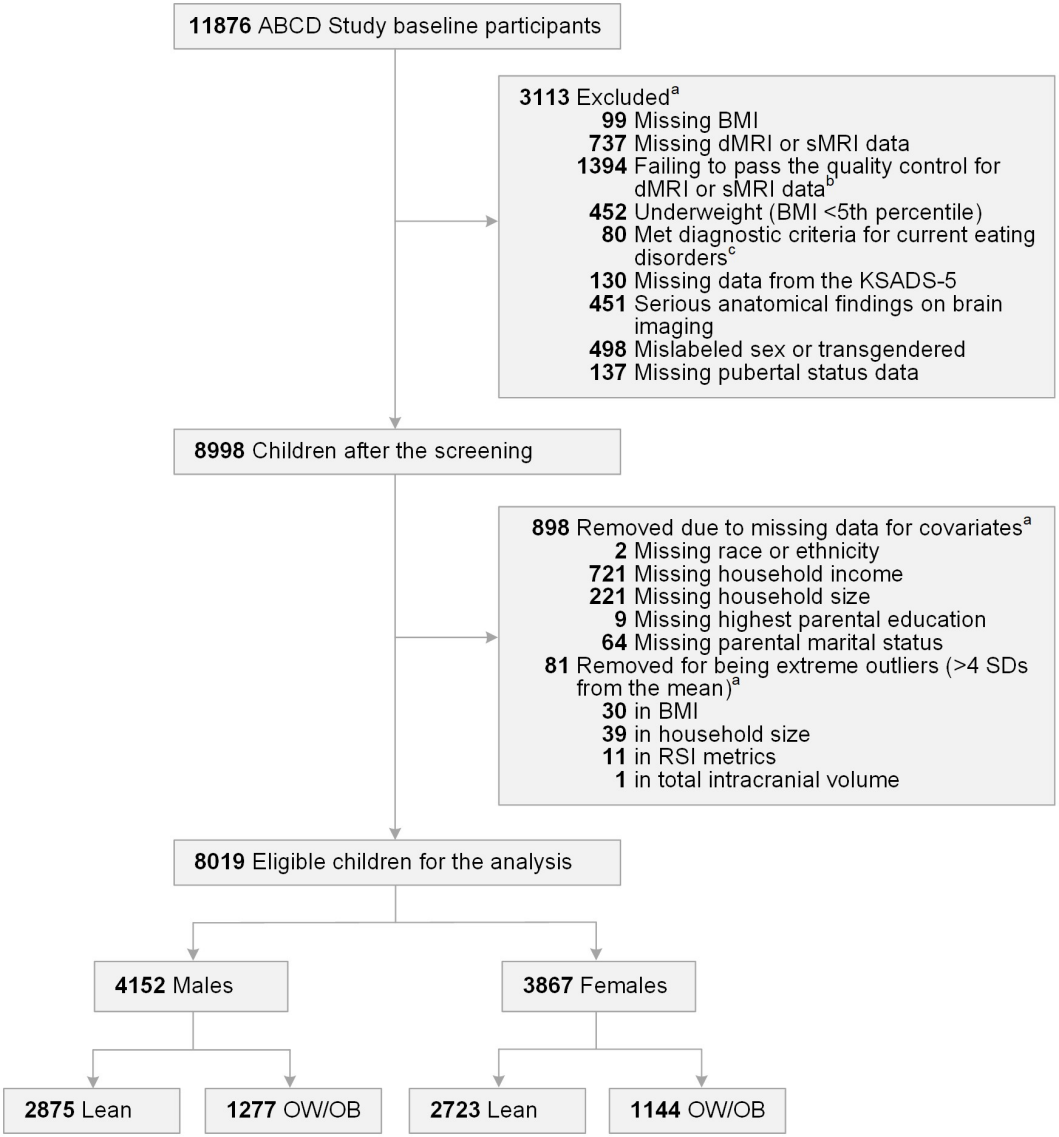
Flow chart of study participants. The study initially assessed 11876 children aged 9 to 10 years recruited from the ABCD Study. After applying the screening and eligibility criteria, data of 8019 children were used in our analysis. ^a^ Some participants met more than one criterion. ^b^ Data that did not pass the ABCD quality control [27] were excluded. ^c^ Parent responses to the KSADS-5 criteria were used for the diagnosis of eating disorders. ABCD = Adolescent Brain and Cognitive Development; BMI = body mass index; dMRI = diffusion magnetic resonance imaging; sMRI = structural magnetic resonance imaging; KSADS-5 = Kiddie Schedule for Affective Disorders and Schizophrenia for the Diagnostic and Statistical Manual of Mental Disorders-5 (DSM-5); SD = standard deviation; RSI = restriction spectrum imaging; OW = overweight; OB = obese.

The data included in the current analysis were from the ABCD Study data release 4.0 (https://data-archive.nimh.nih.gov/abcd). The ABCD Study protocol was approved by the institutional review board (IRB) at each ABCD data collection site, with a central IRB approval at the University of California, San Diego. Parents/guardians provided written informed consent, and assent to participate was obtained from the children. A detailed description of the enrollment procedures of the ABCD Study is provided on the ABCD website (https://abcdstudy.org) and elsewhere [24–26]. Our hypotheses were focused on clarifying the association between overweight/obesity status and brain white matter in a predominantly preadolescent group therefore our analyses included an examination of baseline data (ages 9 to 10 years) from this cohort. Among 11876 children who completed the first wave of the ABCD Study from 2016 to 2018, we excluded children who met any of the following criteria (Fig 1): missing BMI data; missing either diffusion or structural magnetic resonance imaging (MRI) data which passed the quality control procedures (described in Hagler et al. [27]); underweight (BMI less than 5th percentile for age and sex on the Centers for Disease Control and Prevention [CDC] growth charts for the United States [28, 29]); diagnosed with a current eating disorder based on parent responses to the Kiddie Schedule for Affective Disorders and Schizophrenia for the DSM-5 (KSADS-5); clinically significant anatomical findings on brain imaging; mislabeled sex assigned at birth, or transgendered; or missing data for covariates. We also removed extreme outliers (i.e., values beyond four standard deviations [SD] from the mean) in continuous variables to avoid potential measurement errors. The final sample included 8019 children, and the characteristics of these children are summarized in Table 1.

**Table 1:** Sample characteristics.

Characteristic	All	Males	Females
	(*n* = 8019)	(*n* = 4152)	(*n* = 3867)
Age, years	9.9 ± 0.6	9.9 ± 0.6	9.9 ± 0.6
BMI			
5th to 85th percentile	5598 (69.8)	2875 (69.2)	2723 (70.4)
≥85th percentile	2421 (30.2)	1277 (30.8)	1144 (29.6)
Mean, kg/m^2^	18.6 ± 3.6	18.5 ± 3.5	18.7 ± 3.7
Race or ethnicity			
Asian	141 (1.8)	70 (1.7)	71 (1.8)
Black	950 (11.8)	463 (11.2)	487 (12.6)
Hispanic	1507 (18.8)	788 (19.0)	719 (18.6)
White	4597 (57.3)	2414 (58.1)	2183 (56.5)
Other	824 (10.3)	417 (10.0)	407 (10.5)
Income-to-needs ratio	3.9 ± 2.4	3.9 ± 2.4	3.8 ± 2.5
Highest parental education			
≤High school diploma	819 (10.2)	409 (9.9)	410 (10.6)
Some college	1996 (24.9)	1044 (25.1)	952 (24.6)
Bachelor’s degree	2170 (27.1)	1139 (27.4)	1031 (26.7)
Postgraduate degree	3034 (37.8)	1560 (37.6)	1474 (38.1)
Parent marital status			
Married	5759 (71.8)	3014 (72.6)	2745 (71.0)
Widowed	58 (0.7)	32 (0.8)	26 (0.7)
Divorced	712 (8.9)	376 (9.1)	336 (8.7)
Separated	268 (3.3)	130 (3.1)	138 (3.6)
Never married	822 (10.3)	396 (9.5)	426 (11.0)
Living with a partner	400 (5.0)	204 (4.9)	196 (5.1)
Pubertal status^a^	1.9 ± 0.7	1.6 ± 0.5	2.2 ± 0.8

Data are presented as mean ± standard deviation (SD) or *n* (%).

^a^ Pubertal (Tanner) staging categories were assessed using the Pubertal Development Scale (1 = Pre, 2 = Early, 3 = Mid, 4 = Late, 5 = Post) [30]. The scores that were averaged across caregiver and child reports are presented.

BMI = body mass index.

### Body mass index (BMI) class

BMI was calculated based on children’s weight and height, which were measured up to three times and averaged. The measurements were taken in light clothing and without shoes. Children were divided into two groups according to their BMI percentiles for age and sex on CDC growth charts for the United States [28, 29] as follows: (1) lean (5th to 85th percentile, Lean group), and (2) overweight or obese (≥85th percentile, OW/OB group).

### Magnetic resonance imaging (MRI) data acquisition

MRI scans were performed on 3T scanner platforms with a 32-channel head coil. High-resolution multi-shell diffusion-weighted images (DWIs) were acquired at 1.7 mm isotropic resolution. For all DWI acquisitions, the multiband echo-planar imaging (EPI) was used with the following parameters: seven b = 0 s/mm^2^ frames, four b-values (six directions at b = 500 s/mm^2^, 15 directions at b = 1000 s/mm^2^, 15 directions at b = 2000 s/mm^2^, and 60 directions at b = 3000 s/mm^2^), and multiband acceleration factor = 3. A field map for each DWI was also obtained for B0 distortion correction. For anatomical reference, T1-weighted images were collected at 1 mm isotropic resolution using the three-dimensional magnetization-prepared rapid acquisition gradient-echo (3D-MPRAGE) pulse sequence. The acquisition parameters for diffusion-weighted and structural images were optimized and harmonized across sites [26, 27].

### Image preprocessing and restriction spectrum imaging (RSI)

The DWI data were preprocessed by the ABCD Data Analysis and Informatics Center (DAIC) using the pipeline described in Hagler et al. [27]. In brief, each DWI was corrected for eddy current, motion, B0 distortion, and gradient nonlinearity distortion. Images were then resampled into rigid-body alignment with an atlas brain with 1.7 mm isotropic resolution.

The RSI model was fitted to the preprocessed DWIs on a voxel-wise basis [27]. RSI separated the fraction of restricted diffusion from the hindered diffusion, based on their intrinsic diffusion properties of separable pools of water within the brain [21]. The diffusion signal was modeled as mixtures of spherical harmonic basis functions. The restricted directional diffusion was derived from the norm of second- and fourth-order spherical harmonic coefficients of the restricted fraction. This feature reflects directional (anisotropic) water diffusion within the intracellular spaces. An increase in restricted directional diffusion has been known to reflect several microstructural properties of white matter, including increased myelination, axonal integrity/coherence, and/or neurite density [31].

White matter tracts were labeled using AtlasTrack, a probabilistic atlas-based method to automatically segment white matter tracts [32]. We chose the fimbria-fornix in the limbic system as our primary region of interest based on previous studies suggesting that this region is highly susceptible to overweight/obesity [8–11]. As an exploratory purpose, we also investigated other limbic white matter tracts, including: cingulate cingulum; parahippocampal cingulum; anterior thalamic radiation; and uncinate. On the basis of previous literature, we expected lower fimbria-fornix integrity in children with overweight/obesity. For all white matter tracts, mean RSI metrics averaged for the bilateral hemispheres were standardized based on the mean and standard deviation of the study sample for the subsequent analysis.

### Aerobic physical activity

The ABCD protocol assessed aerobic physical activity by self-report [33]. Children reported the number of days in a week that they spent time for a total of at least 60 minutes per day in physical activity that increased their heart rate and made them breathe hard some of the time.

### Statistical analysis

We used the mixed-effects models (mixed command in Stata) to examine the differences in RSI metrics of white matter tracts between the Lean and OW/OB groups. Considering the sexual dimorphism in brain development [34] and the potential sex-dependent effects of overweight/obesity on brain structure and function [2, 3, 35], we conducted the analysis stratified by sex assigned at birth. We also tested the relationship between the number of days of aerobic physical activity in a week and RSI metrics of white matter tracts altered in the OW/OB group using the mixed-effects linear regression model. In all models, we included the following variables as covariates: age, race or ethnicity (Asian, Black, Hispanic, White, other), income-to-needs ratio, highest parental education (≤high school diploma, some college, bachelor’s degree, postgraduate degree), parental marital status (married, widowed, divorced, separated, never married, living with a partner), pubertal (Tanner) status (the average score of caregiver and child reports on the Pubertal Development Scale [30]), and total intracranial volume. Income-to-needs ratio was calculated as the mid-point of the household income band divided by the 2017 poverty threshold developed by the US Department of Health and Human Services [36]. Calculation of the variance inflation factor (VIF) demonstrated no evidence of significant multicollinearity among the covariates in any of the models (VIF < 2). The mixed-effects model can accommodate potential within-cluster correlations in the data by incorporating nested random effects. Thus, we included family ID, scanner serial number, and site as nested random effects in all models to account for the inherent clustering caused by the multilevel data structure of the ABCD Study data (e.g., large numbers of siblings and multiple scanners within study sites). The effect of family was nested in scanner serial number, and scanner serial number was nested in site, as has been recommended [37]. Significance was assessed using a two-tailed *p* <0.05 after Bonferroni correction for multiple comparisons. All statistical analyses were conducted using Stata MP version 16.0 (StataCorp LP, College Station, Texas, US).

As mentioned above, children with transgender identities were not included in our analyses, however the descriptive statistics for children who met our inclusion criteria and identified with transgender identities (*n* = 4) are presented in S1 Table.

## Results

Among 8019 children (mean [SD] age, 9.9 [0.6] years [range: 8.9–11.0 years]; 4152 males [51.8%]; 4597 White [57.3%]), 1277 males (30.8% of males) and 1144 females (29.6% of females) were classified as the OW/OB group (Table 1). The demographic information by the BMI class is summarized in S2 Table. As expected from previous research [38–40], several demographic variables and pubertal status were related to the BMI class, supporting the use of these variables as covariates in our statistical analysis.

### The association between the BMI class and white matter integrity

**Fig 2:**
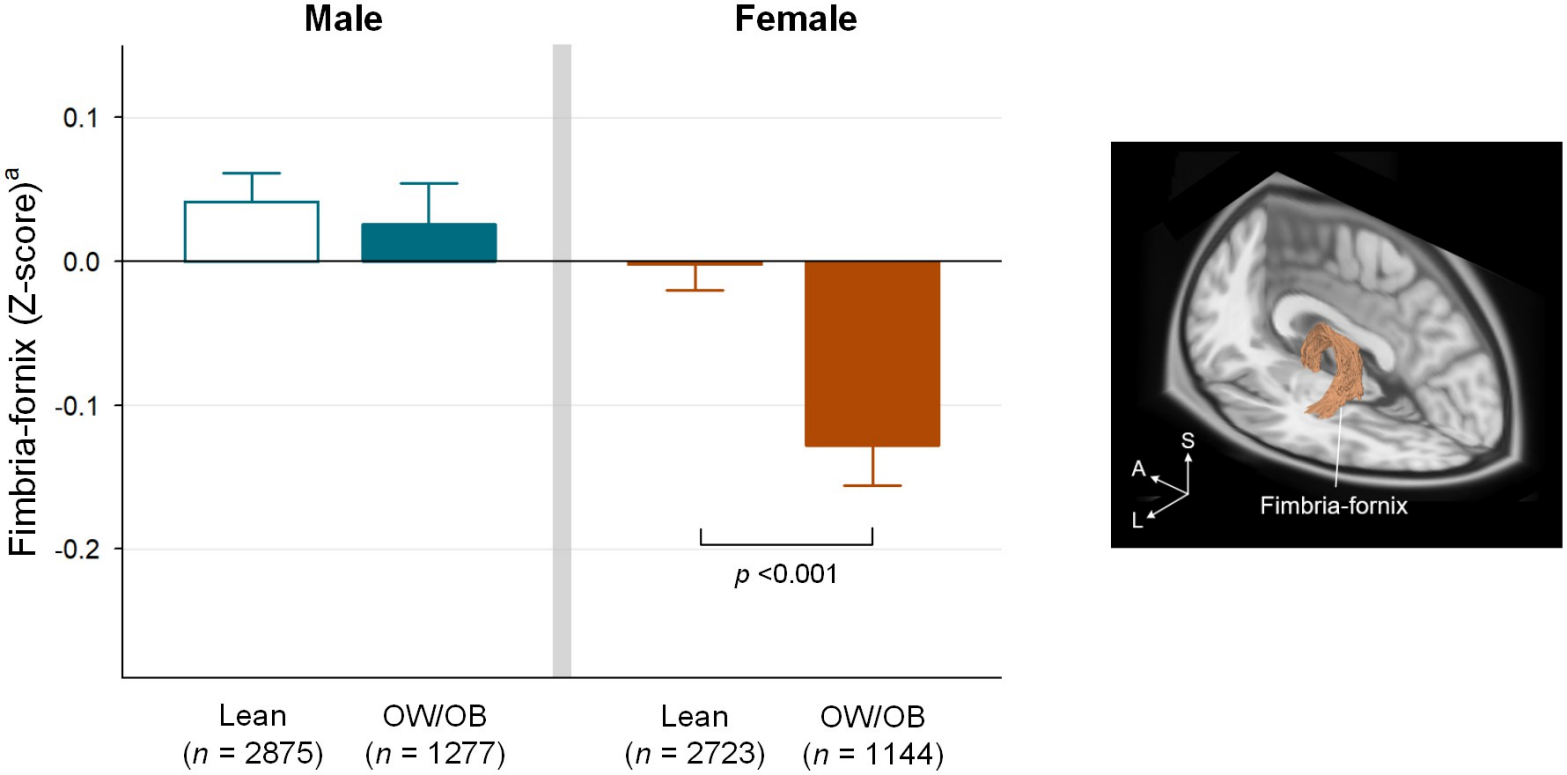
Associations between the BMI class and fimbria-fornix integrity. In females, the OW/OB group showed lower restricted directional diffusion (*R*^2^ = 0.012, *B* = -0.14 [95% confidence interval = -0.19 to -0.08], *z* = -4.85, *p* <0.001) in the fimbria-fornix than the Lean group, while this relationship was not significant in males (*R*^2^ = 0.010, *B* = -0.01 [95% confidence interval = -0.08 to 0.05], *z* = -0.42, *p* = 1.00). The mixed-effects model was used to compare RSI metrics between the BMI groups in each sex, including the following variables: age, race or ethnicity, income-to-needs ratio, highest parental education level, parental marital status, pubertal status, and total intracranial volume as fixed effects; and family ID, scanner serial number, and site as random effects. Each column and error bar represents the mean and corresponding standard errors, respectively. The location of the fimbria-fornix mounted on the T1-weighted slices is provided for reference. ^a^ RSI metrics that were adjusted for age, race or ethnicity, income-to-needs ratio, highest parental education level, parental marital status, pubertal status, and total intracranial volume are presented. BMI = body mass index; OW = overweight; OB = obese; RSI = restriction spectrum imaging; A = anterior; S = superior; L = left.

Females in the OW/OB group showed lower fimbria-fornix integrity, as measured by RSI, compared to those in the Lean group (restricted directional diffusion, *R*^2^ = 0.012, *B* = -0.14 [95% confidence interval = -0.19 to -0.08], *z* = -4.85, *p* <0.001)(Fig 2). This difference was not significant in males (*R*^2^ = 0.010, *B* = -0.01 [95% confidence interval = -0.08 to 0.05], *z* = -0.42, *p* = 1.00).

No other limbic white matter tracts demonstrated a significant difference in RSI-derived integrity measure between the OW/OB and Lean groups in both males and females (Table 2).

**Table 2:** Associations between the BMI class and RSI-derived microstructural integrity measure in each limbic white matter tract.

	Males				Females			
	*R* ^2^	*B* (95% CI)	*z*	*p*	*R* ^2^	*B* (95% CI)	*z*	*p*
Fimbria-fornix	0.010	-0.01 (-0.08, 0.05)	-0.42	1.00	0.012	-0.14 (-0.19, -0.08)	-4.85	<0.001
Cingulate cingulum	0.043	-0.05 (-0.11, 0.01)	-1.70	0.45	0.027	-0.05 (-0.12, 0.01)	-1.66	0.48
Parahippocampal cingulum	0.066	-0.03 (-0.08, 0.02)	-1.13	1.00	0.087	-0.05 (-0.10, 0.004)	-1.81	0.35
Anterior thalamic radiation	0.008	-0.04 (-0.09, 0.02)	-1.38	0.84	0.007	-0.05 (-0.13, 0.02)	-1.53	0.63
Uncinate	0.078	-0.05 (-0.11, 0.005)	-1.78	0.37	0.073	-0.05 (-0.11, 0.002)	-1.87	0.30

All *p* values were Bonferroni corrected for multiple comparisons.

BMI = body mass index; RSI = restriction spectrum imaging; CI = confidence interval.

As a supplementary analysis, we also examined the relationship between the tendency towards overweight/obesity as measured by anthropometric indicators (BMI and BMI percentile for age and sex) and integrity measures in limbic white matter tracts in the OW/OB group. The mixed-effects linear regression analyses with the same covariates and random effects as the above analyses showed no significant correlations in both sexes (S3 and S4 Tables).

### The association between aerobic physical activity and white matter integrity in children with OW/OB

**Fig 3:**
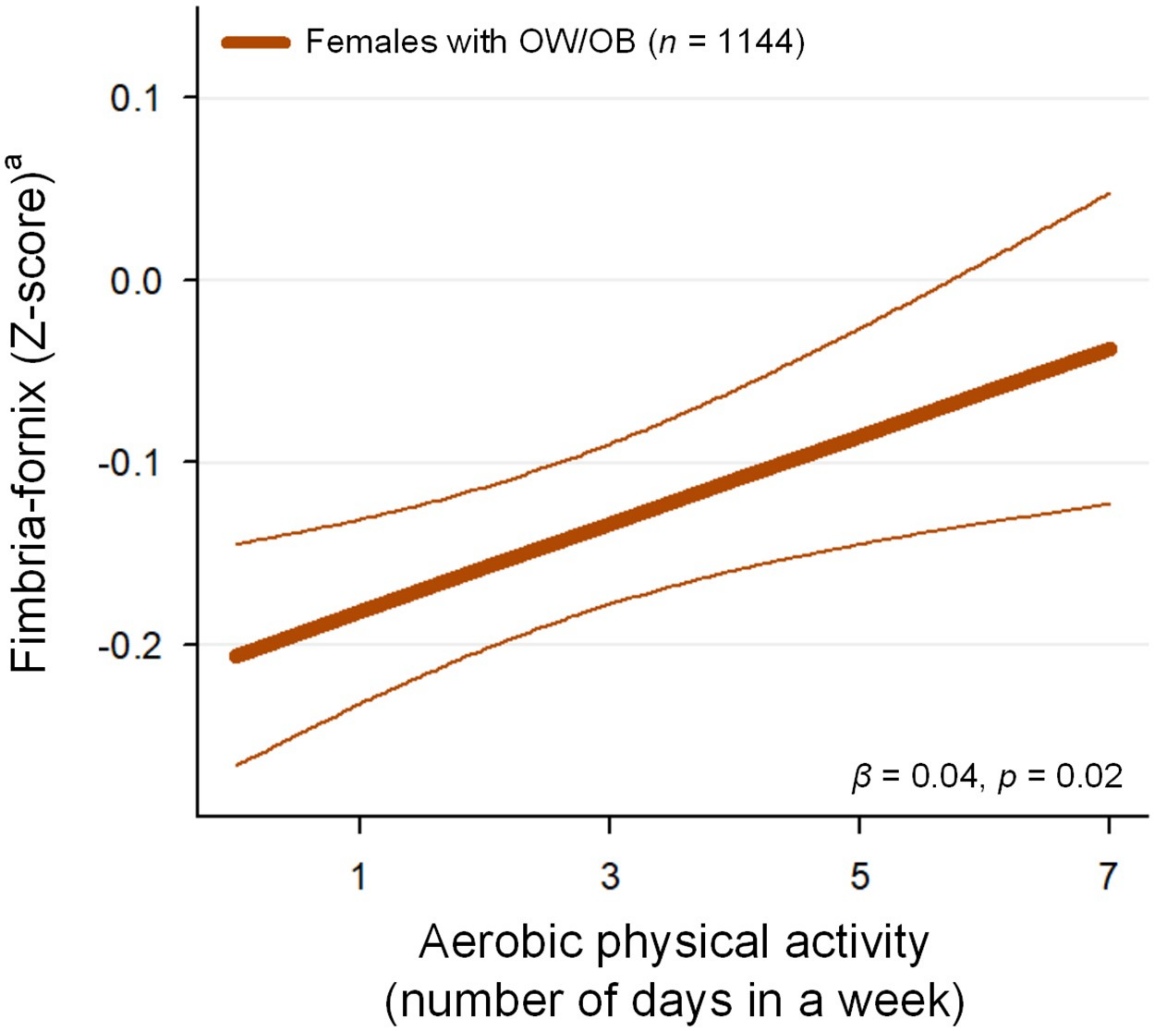
Associations between aerobic physical activity and fimbria-fornix integrity in females with OW/OB. The number of days of aerobic physical activity in a week was positively associated with restricted directional diffusion of the fimbria-fornix in females with OW/OB (*R*^2^ = 0.009, *β* = 0.04 [95% confidence interval = 0.01 to 0.07], *z* = 2.36, *p* = 0.02). The mixed-effects linear regression model was used to test the relationship between aerobic physical activity and RSI metrics, including the following variables: age, race or ethnicity, income-to-needs ratio, highest parental education level, parental marital status, pubertal status, and total intracranial volume as fixed effects; and family ID, scanner serial number, and site as random effects. Bold and thin solid lines represent the regression line and its 95% confidence interval, respectively. ^a^ RSI metrics that were adjusted for age, race or ethnicity, income-to-needs ratio, highest parental education level, parental marital status, pubertal status, and total intracranial volume are presented. ^b^ We assessed the number of days in a week that the children spent time for a total of at least 60 minutes per day in physical activities that increased their heart rate and made them breathe hard some of the time. OW = overweight; OB = obesity or obese; RSI = restriction spectrum imaging.

In females with OW/OB, a higher number of days of aerobic physical activity per week was associated with a greater fimbria-fornix integrity (restricted directional diffusion, *R*^2^ = 0.009, *β* = 0.04 [95% confidence interval = 0.01 to 0.07], *z* = 2.36, *p* = 0.02)(Fig 3). This relationship was not significant in males with OW/OB (*R*^2^ = 0.010, *β* = -0.02 [95% confidence interval = -0.06 to 0.03], *z* = -0.63, *p* = 0.53). Of note, there was no significant relationship between BMI and the number of days of aerobic physical activity in a week in females in the OW/OB group (*R*^2^ = 0.125, *β* = -0.005 [95% confidence interval = -0.05 to 0.04], *z* = -0.21, *p* = 0.83), suggesting that the significant association between aerobic physical activity and fimbria-fornix integrity probably was not simply due to the differences in BMI across aerobic physical activity levels.

## Discussion

Using a large-scale database and an advanced diffusion modeling technique called RSI, we demonstrated that there are relationships among weight status, aerobic physical activity, and fimbria-fornix microstructural integrity in preadolescent females. Decreased fimbria-fornix microstructural integrity in female children with overweight/obesity reflects reduced axonal/dendritic density and less efficient neural processing between the hippocampus and other brain regions in this population. From previous animal and adult human studies, a high vulnerability of the fimbria-fornix to overweight/obesity and its vascular/inflammatory cascades has been reported [8–11], suggesting a directional effect of microstructural integrity as a consequence of weight gain. It has also been demonstrated that reduced integrity of this brain region can increase food intake and subsequent weight gain in animal models [12]. Our results add cross-sectional evidence for these relationships in preadolescent females. To clarify the directional order of our results, longitudinal data analyses are warranted.

Further, given the important role of the fimbria-fornix in learning, memory, and emotional processing, the observed white matter microstructural alterations suggest that childhood overweight/obesity may be associated with different developmental trajectories of the hippocampal networks and related cognitive and emotional functions. Collectively, our findings may suggest the need for proactive and effective intervention against overweight/obesity in preadolescent females.

While the current study design cannot precisely isolate the causal factors for the sex-specific association of overweight/obesity and fimbria-fornix integrity, there are at least four potential candidates, including differences in pubertal development between males and females, X chromosome dosage, and sexual dimorphism in brain development as well as in the brain’s reward system, each of which may have contributed to some extent. First, though the exact mechanisms are largely unknown, it has been reported that pubertal development may increase the risk of metabolic complications, such as increased insulin resistance, low-grade inflammation, oxidative stress, and vascular dysfunctions [41–44], in children and adolescents with overweight/obesity, which, in turn, may confer white matter microstructural impairments [45]. Females with overweight/obesity in our sample showed a relatively faster pubertal development than males with overweight/obesity, with most females with overweight/obesity being early to mid stages of puberty, while a large portion of males with overweight/obesity being pre- to early stages of puberty (S2 Table). Thus, we tentatively speculate that differences in pubertal status between females and males may partly have influenced the observed sex-specific white matter alterations.

In addition to the role that sex differences in pubertal development may play in affecting the fimbria-fornix integrity, it is also possible that genetic components related to sex differences in metabolism may underlie the observed sex differences. Most genes on one of the two X chromosomes in females are silenced to compensate for gene dosage differences between females (XX) and males (XY). However, some of these genes escape the silencing process and are expressed at higher levels in females than in males. Previous rodent studies [46–48] have revealed that X-linked genes involved in lipid metabolism, such as *Kdm5c* and *Eif2s3x*, are critical for sex differences in obesity and metabolism. In these previous studies, the modulation of genomic dosage of these genes in female mice has resulted in adipose tissue expansion as well as increased obesity-related metabolic disturbances, including hyperinsulinemia and dyslipidemia, all of which may affect white matter microstructure [45]. These possible influences of genetic components on white matter could be a contributing factor affecting our results, considering that children in our sample were mainly pre-pubertal or entering puberty.

Another possibility to influence the sex-dependent result is the differences in white matter development between males and females. A recent study on brain development indicated that boys aged 8 to 11 years display more dramatic growth of the fimbria-fornix than girls [36]. This may have made us less likely to detect the overweight/obesity-related microstructural alterations of the fimbria-fornix in males and may play a role in our findings.

The aforementioned speculations on our sex-specific results are based on the assumption that overweight/obesity influences white matter microstructure. However, the opposite direction (e.g., sex-dependent brain mechanisms underlying weight gain) should also be noted. Previous studies have reported sex differences in the involvement of the limbic circuits in reward-related behavior. For example, an increase in BMI in females may be related to more prominent alterations in core reward network regions associated with emotional regulation than in males [49, 50]. Genetic contribution to the sex-dependent development in the reward circuit has also been reported [51]. These studies support that sex-specific brain alterations preceding weight gain may contribute to our results.

We were not able to detect significant associations between anthropometric indicators (BMI and BMI percentile for age and sex) and white matter integrity within the OW/OB group in both sexes, suggesting that the integrity in limbic white matter tracts is not different depending on the levels of overweight/obesity. However, it should be acknowledged that BMI may be poor in differentiating between body fat and lean mass. It may be important to assess other indices of obesity, apart from BMI, including body fat percentage and central/visceral adiposity, in future studies validating the relationships between overweight/obesity and brain abnormalities.

Notably, we also found a positive association between the number of days of aerobic physical activity in a week and fimbria-fornix integrity in females with overweight/obesity, highlighting the potential therapeutic role of aerobic physical activity for children with overweight/obesity. The current finding supports the previously reported evidence for the effects of aerobic physical activity on restoring white matter integrity. A number of studies have demonstrated that aerobic physical activity/exercise effectively reduces vascular and inflammatory stress [18, 19], thereby recovering brain plasticity and myelination of the hippocampus and adjacent brain regions [52].

However, one other explanation may account for the current findings, and that is the effect of the low frequency of aerobic physical activity on the reduction of white matter integrity. Specifically, previous studies reported that limited movement may suppress the maturation of the hippocampal circuits by decreasing circulating neurotrophins such as BDNF [53]. Future intervention studies could directly examine the effects of aerobic physical activity on the fimbria-fornix in children with overweight/obesity.

Further, the abovementioned association between aerobic physical activity and fimbria-fornix integrity was found only in females with overweight/obesity and not in males with overweight/obesity. The observed difference may be due to baseline white matter characteristics. In detail, females with overweight/obesity had reduced fimbria-fornix microstructural integrity, while males with overweight/obesity did not, and thus females with overweight/obesity may show greater benefit from aerobic physical activity. This speculation is supported by a previous study in children aged 7 to 11 years [54], which reported positive associations between cardiorespiratory fitness and white matter volume only in children with white matter reductions.

### Limitations and future directions

The cross-sectional design of the current study does not allow us to ascertain the direction of causality. Nevertheless, our findings, along with the existing literature, have public health messages suggesting the overweight/obesity-associated brain alterations in children, as well as a potential intervention to minimize these alterations. A major strength of our study was its sample size, the largest sample used to date to examine the associations between overweight/obesity and fimbria-fornix microstructure in preadolescent children. This enabled us to detect the relatively small effects (0.9–1.2% of the variance), as observed in other neuroimaging studies with large sample sizes [55]. Given the observed effect sizes, the clinical significance of our findings may not be quite profound at this moment. Nonetheless, the current findings may provide meaningful insight into brain health, considering that potential effects of overweight/obesity on white matter microstructure may accumulate over time [7]. The results should be interpreted with caution since it is possible that the relationships among overweight/obesity, aerobic physical activity, and white matter integrity are mediated by variables not included in this study, such as screen time [56, 57] and sleep duration and disturbances [57, 58]. An additional limitation of our study is that the types and intensity of aerobic physical activity were not classified. Our assessment was based on the number of days in a week that children engaged in aerobic physical activity for a total of at least 60 minutes. Future studies with a more detailed examination of the nature of aerobic physical activity would help clarify advanced clinical guidelines.

As noted, our findings of reduced fimbria-fornix integrity in females with overweight/obesity suggest that typical patterns of fimbria-fornix development may be disrupted in this population and warrant future studies to examine the clinical relevance of our findings. Previous studies have reported that decreased fimbria-fornix integrity is associated with the development of cognitive and emotional disturbances [13, 14, 59]. Intriguingly, low longitudinal academic achievement and the development of psychological symptoms in children and adolescents with overweight/obesity have often been more prominent in girls than in boys [2, 3]. While there is a widely acknowledged impact of sociocultural and interpersonal factors, including weight stigma, lower self-esteem, and body image concerns, on psychological problems in female children with overweight/obesity [15], our results, together with the evidence that reduced fimbria-fornix integrity may cause hippocampal volume decrease [60], might shed light on the brain-level mechanisms underlie the sex-specific development of such neuropsychiatric conditions.

While our findings serve as a base point for the female-specific association between overweight/obesity and limbic white matter alterations in preadolescent children, whether this association would continue or be mediated by dynamic neurologic and physiologic changes during adolescence should be explored in future longitudinal analyses. In addition, elucidating the directional relationship between overweight/obesity and the brain would provide useful insights for the management as well as prevention of overweight/obesity. As an ongoing longitudinal study, the ABCD Study will provide an opportunity to address such research questions.

### Conclusion

To our knowledge, this is the first study providing cross-sectional evidence of reduced microstructural integrity of the fimbria-fornix in preadolescent females with overweight/obesity. Our findings suggest sex-related differences in the developmental trajectories of brain regions involved in cognitive and emotional processing in childhood overweight/obesity. Also, we first showed a positive association between aerobic physical activity and microstructural integrity of the fimbria-fornix in females with overweight/obesity, providing insights for future intervention studies examining neurobiological roles of aerobic physical activity on the overweight/obesity-related brain alterations in children.

## Supporting information

S1 Table Descriptive statistics for children with transgender identities. (PDF)

S2 Table Demographic characteristics of participants across the BMI class. (PDF)

S3 Table Associations between BMI and RSI-derived microstructural integrity measure in each limbic white matter tract in the OW/OB group. (PDF)

S4 Table Associations between BMI percentile and RSI-derived microstructural integrity measure in each limbic white matter tract in the OW/OB group. (PDF)
