# Supplementary material for: Overweight/Obesity-related microstructural alterations of the fimbria-fornix in the ABCD study: The role of aerobic physical activity
Source: PLoS One. 2023 Jul 12;18(7):e0287682. doi: 10.1371/journal.pone.0287682 (PMC10337868; doi:10.1371/journal.pone.0287682)
Supplement: S1 Table — (PDF) [file pone.0287682.s001.pdf]

S1 Table. Descriptive statistics for children with transgender identities.

	Trans male	Trans female	
	OW/OB (*n* = 1)	Lean (*n* = 2)	OW/OB (*n* = 1)
Fimbria-fornix[a], Z-score	0.39	0.64 ± 1.25	0.57
Aerobic physical activity, number of days in a week	3	5.5 ± 0.7	3

Mean ± standard deviations are presented when applicable.

[a] Restriction spectrum imaging (RSI)-derived integrity measures that were adjusted for age, race or ethnicity, income-to-needs ratio, highest parental education level, parental marital status, pubertal status, and total intracranial volume are presented.

OW = overweight; OB = obese.
