# Supplementary material for: Overweight/Obesity-related microstructural alterations of the fimbria-fornix in the ABCD study: The role of aerobic physical activity
Source: PLoS One. 2023 Jul 12;18(7):e0287682. doi: 10.1371/journal.pone.0287682 (PMC10337868; doi:10.1371/journal.pone.0287682)
Supplement: S2 Table — (PDF) [file pone.0287682.s002.pdf]

S2 Table. Demographic characteristics of participants across the BMI class.

Characteristic	Males		Females	
	Lean (*n* = 2875)	OW/OB (*n* = 1277)	Lean (*n* = 2723)	OW/OB (*n* = 1144)
Age, years	9.9 ± 0.6	10.0 ± 0.6	9.9 ± 0.6	9.9 ± 0.6
Race or ethnicity				
Asian	50 (1.7)	20 (1.6)	59 (2.2)	12 (1.0)
Black	270 (9.4)	193 (15.1)	236 (8.7)	251 (21.9)
Hispanic	411 (14.3)	377 (29.5)	430 (15.8)	289 (25.3)
White	1850 (64.3)	564 (44.2)	1715 (63.0)	468 (40.9)
Other	294 (10.2)	123 (9.6)	283 (10.4)	124 (10.8)
Income-to-needs ratio	4.1 ± 2.4	3.4 ± 2.4	4.1 ± 2.4	3.1 ± 2.4
Highest parental education				
≤High school diploma	215 (7.5)	194 (15.2)	209 (7.7)	201 (17.6)
Some college	642 (22.3)	402 (31.5)	547 (20.1)	405 (35.4)
Bachelor's degree	826 (28.7)	313 (24.5)	784 (28.8)	247 (21.6)
Postgraduate degree	1192 (41.5)	368 (28.8)	1183 (43.4)	291 (25.4)
Parent marital status				
Married	2194 (76.3)	820 (64.2)	2084 (76.5)	661 (57.8)
Widowed	19 (0.7)	13 (1.0)	16 (0.6)	10 (0.9)
Divorced	238 (8.3)	138 (10.8)	214 (7.9)	122 (10.7)
Separated	77 (2.7)	53 (4.2)	83 (3.0)	55 (4.8)
Never married	233 (8.1)	163 (12.8)	225 (8.3)	201 (17.6)
Living with a partner	114 (4.0)	90 (7.0)	101 (3.7)	95 (8.3)
Pubertal status[a]	1.6 ± 0.5	1.7 ± 0.6	2.0 ± 0.8	2.6 ± 0.7

Data are presented as mean ± standard deviation or *n* (%).

[a] Pubertal (Tanner) staging categories were assessed using the Pubertal Development Scale (1 = Pre, 2 = Early, 3 = Mid, 4 = Late, 5 = Post) [1]. The scores that were averaged across caregiver and child reports are presented.

BMI = body mass index; OW = overweight; OB = obese.

Reference for the supporting information

1. Petersen AC, Crockett L, Richards M, Boxer A. A self-report measure of pubertal status: Reliability, validity, and initial norms. J Youth Adolesc. 1988;17(2):117–33. doi: 10.1007/BF01537962.
