# Supplementary material for: Overweight/Obesity-related microstructural alterations of the fimbria-fornix in the ABCD study: The role of aerobic physical activity
Source: PLoS One. 2023 Jul 12;18(7):e0287682. doi: 10.1371/journal.pone.0287682 (PMC10337868; doi:10.1371/journal.pone.0287682)
Supplement: S3 Table — (PDF) [file pone.0287682.s003.pdf]

S3 Table. Associations between BMI and RSI-derived microstructural integrity measure in each limbic white matter tract in the OW/OB group.

	Males in the OW/OB group				Females in the OW/OB group			
	R^2	β (95% CI)	z	p	R^2	β (95% CI)	z	p
Fimbria-fornix	0.011	-0.053 (-0.099, -0.007)	-2.26	0.12	0.007	-0.005 (-0.047, 0.037)	-0.25	1.00
Cingulate cingulum	0.077	0.017 (-0.030, 0.063)	0.70	1.00	0.031	-0.019 (-0.072, 0.034)	-0.70	1.00
Parahippocampal cingulum	0.072	-0.005 (-0.049, 0.039)	-0.22	1.00	0.108	-0.031 (-0.082, 0.019)	-1.22	1.00
Anterior thalamic radiation	0.008	0.015 (-0.031, 0.061)	0.63	1.00	0.011	0.015 (-0.026, 0.055)	0.72	1.00
Uncinate	0.110	0.014 (-0.020, 0.048)	0.81	1.00	0.107	-0.008 (-0.063, 0.047)	-0.29	1.00

All p values were Bonferroni corrected for multiple comparisons.
BMI = body mass index; RSI = restriction spectrum imaging; OW = overweight; OB = obese; CI = confidence interval.
