# Supplementary material for: Overweight/Obesity-related microstructural alterations of the fimbria-fornix in the ABCD study: The role of aerobic physical activity
Source: PLoS One. 2023 Jul 12;18(7):e0287682. doi: 10.1371/journal.pone.0287682 (PMC10337868; doi:10.1371/journal.pone.0287682)
Supplement: S4 Table — (PDF) [file pone.0287682.s004.pdf]

S4 Table. Associations between BMI percentile and RSI-derived microstructural integrity measure in each limbic white matter tract in the OW/OB group.

	Males in the OW/OB group				Females in the OW/OB group			
	R^2	β (95% CI)	z	p	R^2	β (95% CI)	z	p
Fimbria-fornix	0.010	-0.044 (-0.089, -0.0003)	-1.97	0.24	0.007	-0.005 (-0.038, 0.028)	-0.29	1.00
Cingulate cingulum	0.077	0.019 (-0.029, 0.066)	0.78	1.00	0.030	-0.008 (-0.058, 0.042)	-0.31	1.00
Parahippocampal cingulum	0.073	0.001 (-0.035, 0.037)	0.06	1.00	0.108	-0.028 (-0.080, 0.023)	-1.08	1.00
Anterior thalamic radiation	0.008	0.007 (-0.045, 0.060)	0.28	1.00	0.010	0.005 (-0.029, 0.040)	0.29	1.00
Uncinate	0.111	0.013 (-0.024, 0.050)	0.70	1.00	0.107	-0.015 (-0.069, 0.040)	-0.53	1.00

All p values were Bonferroni corrected for multiple comparisons.
BMI = body mass index; RSI = restriction spectrum imaging; OW = overweight; OB = obese; CI = confidence interval.
